# Extracellular volume fraction measured by MOLLI: slow infusion versus bolus

**DOI:** 10.1186/1532-429X-13-S1-P317

**Published:** 2011-02-02

**Authors:** Erik Schelbert, Stephen M Testa, Christopher G Meier, William J Ceyrolles, Joshua Levenson, Alex J Blair, Kathy S Puntil, Peter Kellman, Bobby L Jones, Daniel R Ludwig, Hua Zhong, David Schwartzman, Sanjeev G Shroff, Timothy C Wong

**Affiliations:** 1 University of Pittsburgh, Pittsburgh, PA, USA; 2 Carnegie Mellon University, Pittsburgh, PA, USA; 3 National Heart, Lung, Blood Institute, Bethesda, MD, USA

## Background

Myocardial extracellular volume fraction (Ve) measures from myocardial and blood pool T1 data quantify diffuse fibrosis (Flett, 2010) not detectable by conventional late gadolinium (Gd) enhancement, but require steady state equilibrium between plasma and interstitium. While a bolus with a lengthy infusion produces steady state for Ve measures, it is unclear whether a Gd contrast bolus alone (e.g., Broberg, 2010) is sufficient. Given the relatively slow clearance of Gd, we hypothesized that a simple bolus accurately measures Ve, thus facilitating integration of Ve measurement into CMR workflow routines.

## Methods

In 10 diverse volunteers (ages 20-81, median 33 yr), we compared serial Ve measures from two scans: first, during a constant infusion (0.1 mmol/kg bolus followed by 0.1 mmol/kg diluted in 200mL saline, 200 mL/hr infusion, x1hr), and second, 12-50 min after a bolus (0.2 mmol/kg) on another day. Ve data from a diastolic short axis slice were computed as described by Jerosch-Herold (2008). Steady state during infusion was defined when blood and myocardial T1 varied <5%. We measured T1 on a 1.5 T Siemens scanner using a single-shot modified Look Locker inversion recovery research sequence (MOLLI), similar to Messroghli (2007). Pre-contrast (longer) T1 values were measured with 2 RF pulses and 5+1 heart beat sampling scheme, and post-contrast (shorter) T1 measures used 3 RF pulses and 4+2+1 sampling scheme with at least 1-2 dummy beats separating RF pulses from preceding SSFP readout. At fast or slow heart rates (65-95 bpm) we validated this technique using CuSO4-Agar phantoms with similar T1 and T2 of myocardium/blood, pre- and post-Gd as measured by spin echo inversion recovery (T1) and saturation recovery (T2), 1 k-space line/RF pulse, TR=15 s. MOLLI and spin echo T1 measures (n=210) correlated well (slopes >0.95; p=NS vs. unity) without bias on Bland-Altman plots. Infusion vs. bolus Ve measures (n=205) across subjects were compared with generalized estimating equations with exchangeable correlation matrices for serial measures.

## Results

By infusion, the Ve range was 19.3-29.2% with a SD<=0.9% for repeated measures (n=110) in the 10 subjects. By bolus, the Ve range was 17.4-29.1%, with a SD<=1.3% for the repeated measures (n=95). Serial Ve measures by bolus or infusion did not differ significantly (delta=0.2%, p=0.20).

## Conclusion

Serial Ve measures from either an intravenous bolus or infusion did not differ significantly in a diverse sample of volunteers. Myocardial Ve can be measured reliably and accurately 12-50 minutes after a simple 0.2 mmol/kg bolus.

